# Effect of Graphene Nanoplatelets on the Structure, the Morphology, and the Dielectric Behavior of Low-Density Polyethylene Nanocomposites

**DOI:** 10.3390/ma13214776

**Published:** 2020-10-26

**Authors:** Athena Maniadi, Maria Vamvakaki, Mirela Suchea, Ioan Valentin Tudose, Marian Popescu, Cosmin Romanitan, Cristina Pachiu, Octavian N. Ionescu, Zaharias Viskadourakis, George Kenanakis, Emmanouel Koudoumas

**Affiliations:** 1 Department of Materials Science and Technology, University of Crete, Vassilika Voutes, 70013 Heraklion, Greece; vamvakak@materials.uoc.gr; 2 Center of Materials Technology and Photonics, Hellenic Mediterranean University (Former Technological Educational Institute of Crete), 71004 Heraklion, Greece; tudose_valentin@yahoo.com; 3 National Institute for Research and Development in Microtechnologies (IMT-Bucharest), 126A, Erou Iancu Nicolae Street, 077190 Bucharest, Romania; marian.popescu@imt.ro (M.P.); cosmin.romanitan@imt.ro (C.R.); Cristina.pachiu@imt.ro (C.P.); octavian.ionescu@imt.ro (O.N.I.); 4 Department of Chemistry, University of Crete, Vassilika Voutes, 70013 Heraklion, Greece; 5 Institute of Electronic Structure & Laser (IESL), Foundation for Research and Technology (FORTH) Hellas, 71110 Heraklion, Greece; zach@iesl.forth.gr (Z.V.); gkenanak@iesl.forth.gr (G.K.)

**Keywords:** polymer nanocomposites, low-density polyethylene, graphene, dielectric properties

## Abstract

The incorporation of graphene nanoplatelets (GnPs) within a polymer matrix can play an important role in the physical properties and the functionality of the composite material. Composites consisting of low-density polyethylene (LDPE) and GnPs of different concentrations were developed by mixing GnPs with a molten form of the polymeric matrix. The effect of the GnPs content on the morphological, structural, and electrical properties of the composites were investigated. As shown, graphene presence and its concentration significantly modified the polymer matrix properties, a behavior that can be employed for tailoring its applicability in electrical applications. It was found that the increase of the graphene platelets concentration seems to promote the formation of graphene agglomerates, air gaps, and inhomogeneities, while higher dielectric constant/lower dielectric losses can be achieved.

## 1. Introduction

Polymers have been proved to be suitable matrices for the development of composite materials due to their ease of production and processing, resistance to corrosion, good mechanical properties, and light weight. In polymer-based nanocomposites, the type and size of the filler, the morphology of the filler network, the thermal properties, and the conductivity have been considered to be important variables influencing its final properties [1,2]. Polymers are basically electrical insulators with low dielectric permittivity, properties that make them suitable for particular applications in electrical power systems [3].

Presently, graphene, has become one of the most exciting materials [4], because of its unique properties, including high values of thermal conductivity [5], Young’s modulus [6] and large surface area [7]. These properties make graphene a promising material in many applications including photovoltaic devices, sensors, transparent electrodes, super capacitors, and conducting composites.

Substantial amount of research has been carried out in the field of nanocomposites incorporating graphene, some of them regarding electrical applications. In general, the increasing demand of energy is a technical challenge for the generation, transmission, storage, and distribution energy systems. This challenge often requires contradictory features such as increasing voltage levels in combination with more compact designs in urban environments. This leads to an increased electric stress on the insulation systems, which can be addressed by using insulating materials with tunable properties, as well as high dielectric constant and low losses, especially for electric field grading applications. In this particular research field, the reports in the literature on graphene nanocomposites concern, improvement of conductivity in polyethylene (PE) matrices [8], advanced thermoplastics based on polypropylene matrix composites [9], as well as modification of electrical and mechanical properties of various matrices, including polyvinyl chloride (PVC) [10], acrylonitrile butadiene styrene (ABS) [11] and polylactic acid (PLA) [12,13].

This work concerns nanocomposite materials made using commercial widely available ingredients using methods that can easily undergo direct further scale up, to become compatible with the industrial needs. In particular, in this work, low-density polyethylene (LDPE) was chosen as the basic matrix material for the fabrication of graphene nanocomposites, due to its significant importance in various applications such as squeeze bottles, toys, carrier bags, high frequency insulation, chemical tank linings, heavy duty sacks, general packaging, gas and water pipes [14]. In addition, because of its low procurement cost. LDPE is an important member of the PE (polyethylene) family due to its special electrical behavior, such as low dielectric constant. Because of these properties, PE-based composites can be used as insulating materials. The paper presents the study of GnPs/LDPE composite materials, focusing on the influence of the graphene platelets concentration on the morphology and structure as well as the dielectric properties of the obtained GnPs /LDPE composites. To the best of our knowledge, this is the first report regarding a detailed evolution of the morphology and the structure of LDPE-graphene composites with graphene concentration, using combined results of scanning electron microscopy (SEM), X-ray diffraction (XRD), Raman, and confocal Raman microscopy.

## 2. Experimental Methods

Nanocomposite specimens were fabricated by employing commercially available LDPE from Sigma Aldrich GmbH, Darmstadt, Germany with average molecular weight—MW of 35,000 determined by gel permeation chromatography (GPC) and average numerical mass—Mn 7.700 GPC. Graphene nanoplatelets were provided by EMFUTUR Technologies Ltd., Hillsborough, Spain, with ~5 μm lateral size, 5 nm thickness, and a bulk density of 0.03 to 0.1 g/cm^3^.

The melting method was used since this is rather simple for fabricating polymer nanocomposites consisting of low molecular weight polymer matrices. This method involves melting of the polymer to its viscous form, followed by the addition and the mechanical mixing of the filler within the polymer matrix. Graphene concentration in the composite material was varied in the range 0% (%wt.) up to 5% (%wt.) at a step of 0.5%. For each composition 5 replicas were made and characterized. All the obtained formulations were characterized as follows:

SEM characterization was performed using a (FE-SEM) Nova NanoSEM 630 (FEI Company, Hillsborough, OR, USA), equipped with an energy-dispersive X-ray spectroscopy—EDX detector (EDAX TEAM™, USA), in order to investigate and understand the formation and the structuring of the obtained nanocomposite materials. All samples were examined in the high vacuum mode, low landing energies and fast dwelling time, without any conductive coating in order to preserve the samples in their natural state for further analyses. XRD was also performed using a Rigaku Ultra high-resolution triple axis multiple reflection SmartLab X-ray Diffraction System (Rigaku, Tokyo, Japan). Moreover, Raman analysis was done using a WiTec alpha 300S GmbH Germany system (WiTec., Ulm, Germany), employing an Nd-YAG laser at 532 nm and confocal Raman microscopy (high-resolution confocal Raman imaging). Raman spectra were recorded under ambient conditions, using a 532 nm diode-pumped solid-state laser (Alpha-SNOM 300 S, WiTec. GmbH, Ulm, Germany) with a power of 10% (10 mW) to avoid local drying of the samples. A 20× objective and a 25 μm slit aperture were used in order to obtain quite detailed spectra, while the total acquisition time 0.5, 10 and 20 s (1 s exposure × 20 exposures) and 600 grooves/mm grating was chosen for each Raman spectrum. The only notable difference observed in spectra was a higher overall spectral intensity when the acquisition time increases. Severally spectra, in different points were recorded per sample and a representative typical spectrum for each sample was chosen. The instrument calibrations were performed on a silicon probe prior to measurements to ensure reproducible results. The spectrometer scanning data collection and processing were carried out using the WiTec Project Five software (WiTec GmbH, Ulm, Germany) and appropriate background corrections were performed on all spectra in Origin software (2017, OriginLab, Northampton, MA, USA). The thermal response characterization was studied using a Diamond (Perkin Elmer, Shelton, CT, USA) differential scanning calorimeter (DSC) operated at a scan rate of 10 °C/min in order to characterize the thermal transitions of employed polymer nanocomposites. Samples were prepared inside aluminum shielded pans while an empty aluminum shielded pan was used as a reference. Experimental temperature range was set from ambient to 200 °C. For thermogravimetric analysis (TGA), a Diamond (Perkin Elmer, Shelton, CT, USA) TGA was used under 200 mL/min N_2_ flow and a temperature increment of 20 °C/min. Finally, the electrical characterization of the nanocomposite samples was conducted by means of Broadband Dielectric Spectroscopy (BDS), using a TH2829C LCR precision bridge (HIOKI E.E. Corporation, Nagano, Japan) capable of taking measurements in the frequency range of 20 Hz to 1 MHz.

For the low energy impedance spectroscopy measurements, samples of a rectangular shape were used, with dimensions 10 mm × 10 mm × 0.5 mm. Conductive silver paste was smeared onto both the 10 × 10 mm^2^ areas of the sample in order to form a capacitor. Two metallic pins were attached to the silver paste plates, and these pins were connected to an HIOKI 3532-50 LCR HITESTER (HIOKI E.E. CORPORATION, Nagano, Japan), through its specialized cables. Impedance *Z* and corresponding loss angle δ are simultaneously measured, as a function of frequency, in the frequency range 10 Hz–1 MHz. The presented results are representative for the specific kind of samples.

## 3. Results and Discussion

SEM characterization of samples was performed to investigate the formation and structuring of the composite materials. Nanocomposite materials with higher than 2% graphene content were found to consist of a quite inhomogeneous bulk, formed by different polymerization domains as well as regions with concentrated graphene. In addition, at high graphene concentrations, large graphene agglomerations were observed in some cases, suggesting that a limit of concentration might exist, above which graphene cannot be fully dispersed in the LDPE molten material.

**Figure 1:**
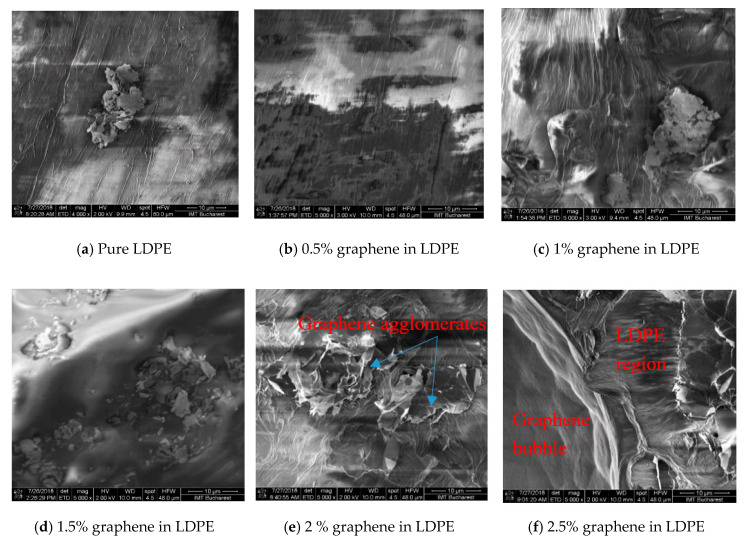
SEM images of samples fabricated at lower concentrations, showing the evolution of materials morphology (×5000 magnification, scale 10 µm).

Figure 1a–f present high resolution (HR) SEM images at a magnification of ×5000 of some of the fabricated samples with different graphene content. As can be seen, increasing graphene concentration leads to larger polymerization domains. At low graphene concentrations a quite good quality, smooth, and uniform composite material morphology appears, comparable with pure LDPE. As the concentration increases the presence of graphene in the composite becomes obvious.

**Figure 2:**
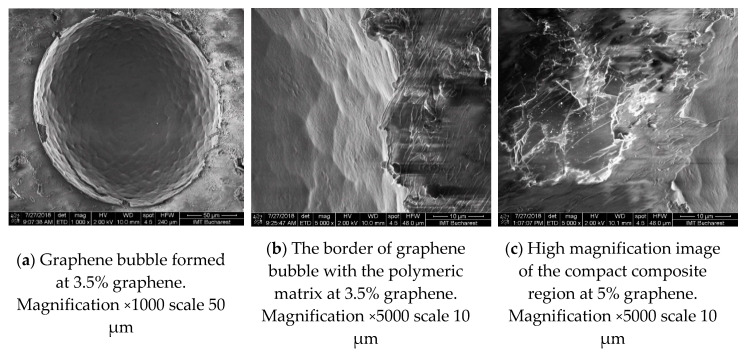
SEM images samples fabricated at higher concentrations, showing the evolution of materials morphology.

At graphene concentrations higher than 2.5%, the structural inhomogeneity is accentuated and a mix of compact and granular regions as well as “graphene bubbles” were observed, as can be seen in Figure 2a–c. In addition, for the same range of graphene concentrations, the “graphene bubbles” density becomes higher and in the intermediary regions wrinkled graphene is observed.

Following the SEM characterization, one can understand the effect of the graphene concentration on the composites structure and morphology. The increase of concentration seems to promote the formation of graphene agglomerates, air gaps, and inhomogeneities being present at the same time. These structural SEM observations, related to the effect of the graphene concentration on the evolution of the bulk morphology, seem to correlate with the dielectric constant measurements, as it will be shown later. Finally, it should be mentioned here that to the best of our knowledge, such detailed morphology study of LDPE-graphene composite and its evolution with graphene concentration has not yet been presented in the literature before.

**Figure 3:**
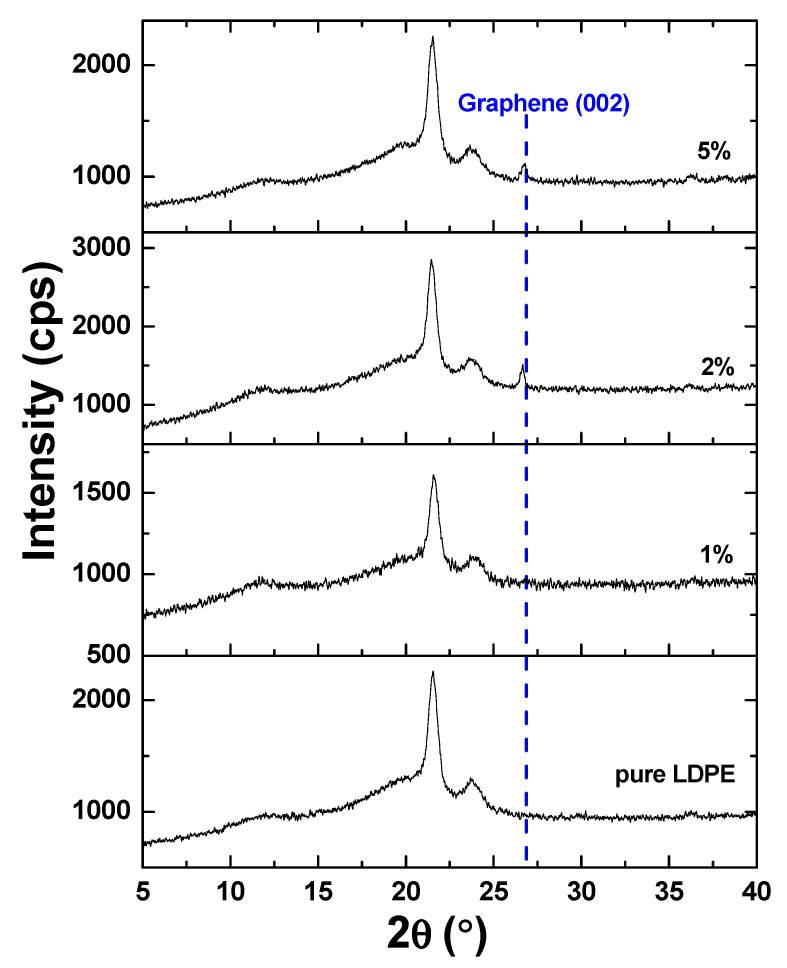
XRD patterns corresponding to pure LDPE and LDPE with different concentrations of graphene. The blue dashed line corresponds to graphene (002).

Structural features for the LDPE composites of different graphene concentrations were further investigated using grazing incidence X-ray diffraction (GI-XRD), where the incidence angle was kept to 0.5°. In order to assess the influence of graphene, the attention was focused in the range of 5°–40°. The results can be seen in Figure 3, which presents typical XRD patterns obtained for three LDPE/graphene composites and pure LDPE, the latter presenting multiple diffraction peaks, typical for LDPEs [15,16,17].

With increasing graphene concentration, the occurrence of the characteristic diffraction peak for carbon around 26° appeared for graphene concentrations higher than 2% graphene. To get the mean crystallite size τ for LDPE and graphene composites, the Scherrer equation [18] was employed:

τ = kλ/(βcosθ)(1)

where k is a shape factor taken equal as 0.9, 2θ is the peak angular position, β is the line broadening at half the maximum intensity and λ is the wavelength of the incident X-ray. In the case of LDPE, the mean crystallite size was found to be around 13.1 nm for all samples checked, pointing out that the crystallinity of LDPE was preserved during the graphene incorporation. In contrast, analyzing the crystallinity of graphene with Scherrer equation, we can observe a modification of the mean crystallite size from 26.1 nm in the intermediate range of graphene concentrations (e.g., 2%) to 40.4 nm at the highest concentration (e.g., 5%). Moreover, when the graphene concentration reaches higher concentrations (e.g., 5%), the corresponding angular position is slightly modified. In particular, the peak position was found to be at 26.76° in the case of intermediate concentrations (e.g., 2%), while, for the highest concentration (5%), the peak position was shifted to 26.58°. According to the Bragg law (i.e., 2dsinθ = λ), which relates the peak position (2θ) by the interplanar distance (d), the latter increased from 3.32 to 3.35 Å.

The obtained results strongly indicate the embedding of the graphene in the LDPE lattice and suggest an enhancement of its crystallinity at high graphene concentration. At the same time, the interlayer distance of the graphene employed becomes closer to the hexagonal graphene one (2H-polytype), for which d_002_ = 3.37 Å, at the highest graphene concentration.

Raman spectra were also recorded, using a 532 nm diode-pumped solid-state laser (Alpha-SNOM 300 S, WiTec. GmbH, Germany) with a power of 10 mW. A 20× objective and a 25 μm slit aperture were used in order to obtain quite detailed spectra, while a total acquisition time of 10 s (1 s exposure × 10 exposures) and 600 grooves/mm grating was chosen for each Raman spectrum. The spectrometer scanning data collection and processing were carried out using the WiTec Project Five software.

**Figure 4:**
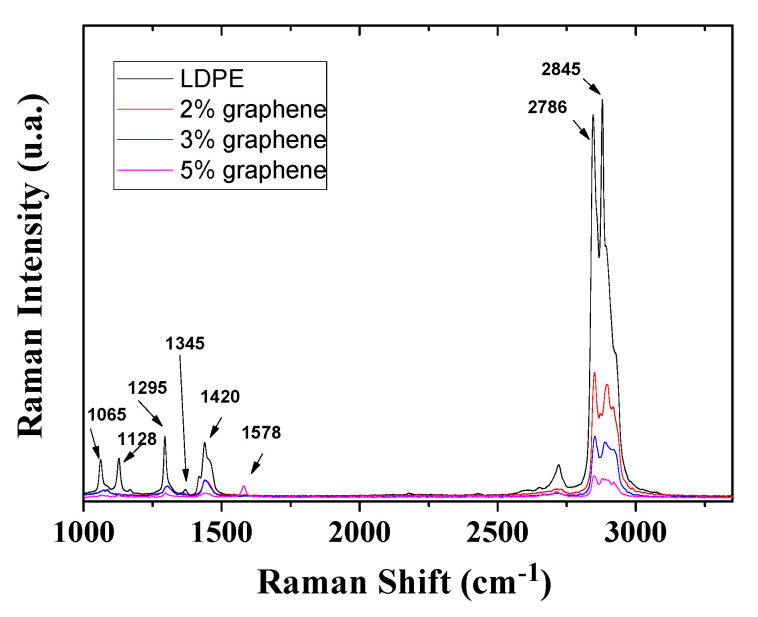
Raman spectra collected on various concentrations on melt-fabricated samples.

Examples of Raman spectra collected on melt-fabricated samples corresponding to various concentrations of graphene are presented in Figure 4, where the graphene and the low-density polyethylene (LDPE) bands are clearly visible and distinct. Specifically, for graphene, the G band is present at about 1578 cm^−1^, the 2D band is at about 2786 cm^−1^, while there is also evidence of the so-called disorder-induced D bands around 1345 cm^−1^. Literature reports similar peaks for graphene, in particular, in pristine-single layer graphene, the G band appearing at 1582 cm^−1^ (graphite) and a 2D band at about 2786 cm^−1^ [19,20]. Moreover, in case of multi-layers graphene, the G peaks are splinted into two peaks, at 1623 cm^−1^ and 1578 cm^−1^ respectively, denoted by the D’, while the I2D/IG ratio increases [21]. Regarding LDPE, its primary peak (C-H stretching) can be seen at 2845 cm^−1^, while peaks near 1420 cm^−1^ (the CH2 bending mode) are also present and are indicators of crystalline and amorphous PE phases. Literature reports similar results for polyethylene, both low and high density, with Sato et al. reporting four (4) peaks at 1465, 1374, 1174 and 897 cm^−1^ [22]. Moreover, Kalman et al., found four (4) peaks near 1080, 1128, 1296 and 1415 cm^−1^ respectively [23]. It can be observed that increasing the concentration of the graphene platelets leads to an enhancement of the graphene D and G modes. At the same time, the C-H bonds are weakening as well as the vibration modes intensities at 2787 cm^−1^ and 2848 cm^−1^.

**Figure 5:**
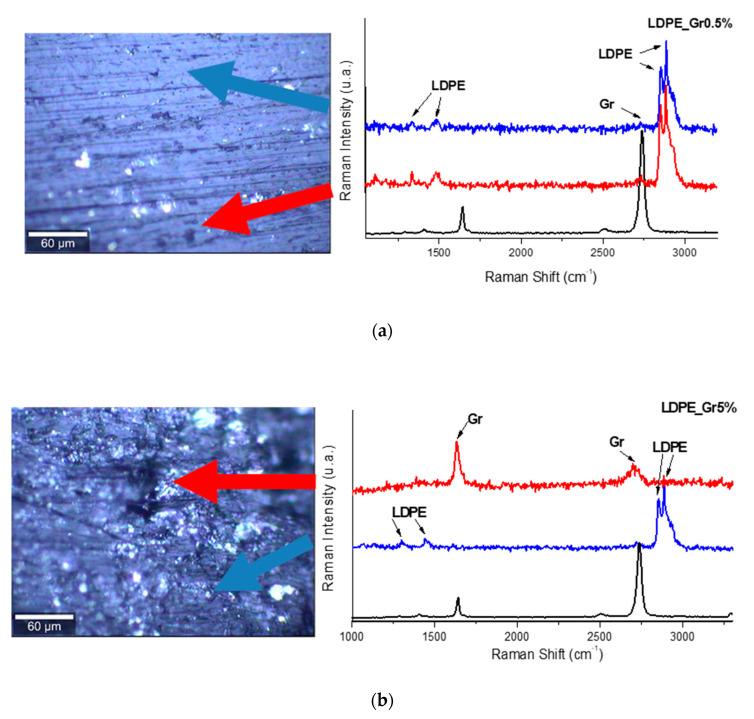
Raman spectra in two different points on the surface for two concentrations: (**a**) 0.5% and (**b**) 5%. The pristine-SL Graphene (black line) are presented as reference for band positions.

Regarding local homogeneity of the composite material, an example is presented in Figure 5, which presents examples of Raman spectra collected two different graphene concentration and two different points of the surface and compared to the spectrum of the graphene as reference. As can be observed, there are regions where the graphene and PE forms very good composite material and some regions where the material is inhomogeneous.

Detailed characterization of the obtained nanocomposites showed that all different batches, fabricated under similar conditions, resulted in more or less similar inhomogeneous materials exhibiting similar properties. These results indicated that the mechanical mix of GnPs in melted LDPE may not be the best method for the preparation of homogeneous GnPs/LDPE nanocomposites, at least for the case of small quantities, as those used in our work. Another approach considered for obtaining GnPs/LDPE nanocomposites formulations in small quantities could be dry mixing of the LDPE polymer pellets with graphene but, as tested, it was almost not possible, since they did not really mix. Similarly, we found out that extruding the two phases resulted in even worst composite material. For proper mixing of the specific materials hot roll milling would be more proper method but it is not easily available for laboratory small scale trials. Since GnPs are not a monodisperse material, functionalization of GnPs would not be a realistic approach to solve the dispersion problems at the mixing in the mold. In any case, more homogeneous materials are expected in larger scale production, since, in this cases mixing can be done more effectively.

**Figure 6:**
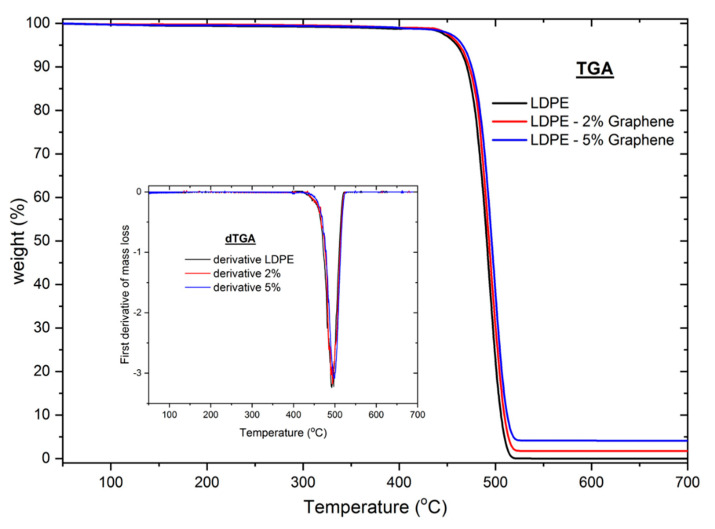
TGA thermal decomposition evolution of pure LDPE, 2% and 5% graphene containing GnPs/LDPE composite materials. Inset: The first derivative of mass loss.

TGA was performed to study the mass loss if a thermal event involves loss of a volatile component. Figure 6 shows some example of the thermal decomposition evolution for pure LDPE, 2% and 5% graphene containing GnPs/LDPE composite materials. As the temperature increases the LDPE depolymerize and the monomers evaporate taking away also small fragments of graphene. The residues at the end of the thermal treatment were evaluated. The obtained values were smaller but proportional with the graphene concentration % in the respective nanocomposite material.

**Figure 7:**
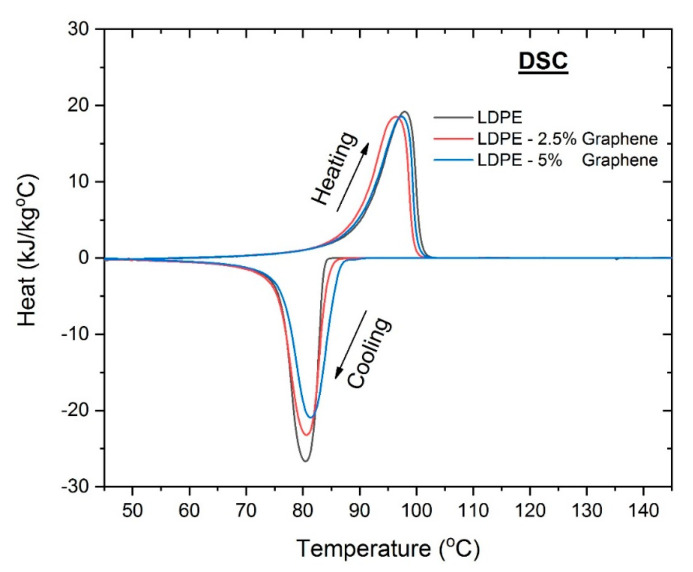
Typical DSC analyses of the 2.5% and 5% graphene containing samples compared with pure LDPE.

To study the phase transitions of the GnPs/LDPE nanocomposites DSC analysis was performed. Two typical DSC analyses of the 2.5% and 5% graphene containing samples compared with pure LDPE are presented in Figure 7.

The melting temperature Tm (°C) for pure LDPE was 80 °C while the crystallization temperature Tc—98 °C. All the composite materials show Tm smaller than the pure material and Tc larger. Tm increases as the GnPs increase and Tc slightly increase when GnPs concentration increased.

**Figure 8:**
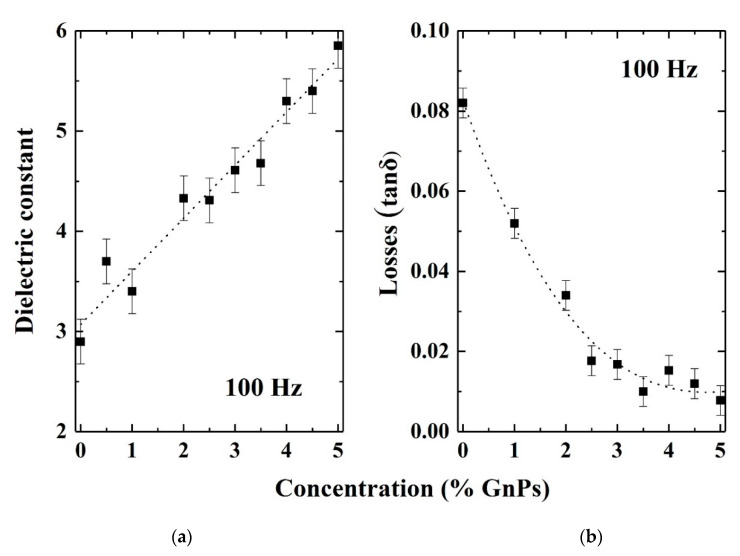
(**a**) Dielectric constant and (**b**) Loss (delta) versus concentration of graphene (% GnPs wt.).

**Figure 9:**
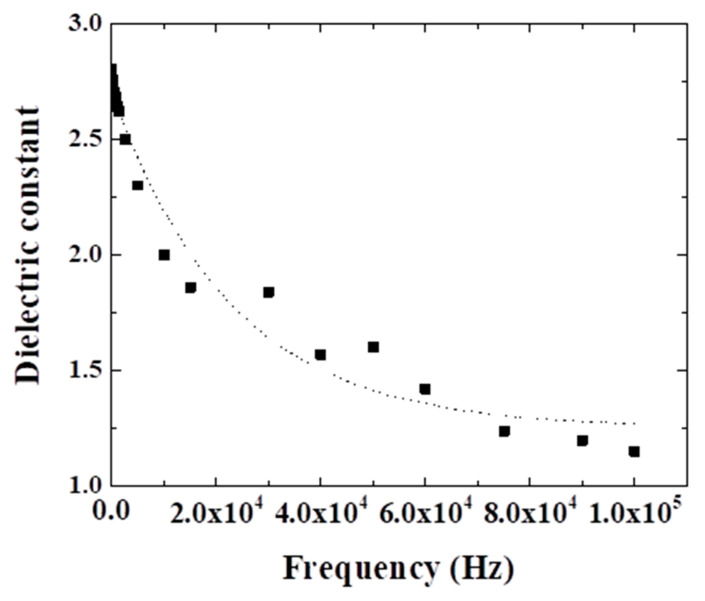
Dielectric constant versus frequency for a sample containing 3% wt. graphene.

Finally, the dielectric behavior of all fabricated samples was investigated using BDS and its dependence on the graphene content within the LDPE matrix was analyzed. Figure 8a,b depicts the influence of the graphene platelets concentration on the dielectric properties of the composite material at a frequency of 100 Hz, the behavior being similar at other frequencies up to 1 MHz. As can be seen, the dielectric constant increases almost linear with graphene concentration, becoming double at a concentration of 5%. In contrast, the respective dielectric losses present a significant reduction, becoming four times smaller at the concentration of 5%. Regarding the variation of the dielectric constant with frequency, this is shown in Figure 9 for a sample containing 3% graphene. As can be observed, increasing frequency results in a quite important decrease on the dielectric constant value.

As expected, the dielectric constant significantly depends on the graphene concentration and the structural characteristics of the material. In particular, the dielectric constant can be strongly influenced by the morphology of the composite, especially if this contains air gaps and agglomerations, as was found out in the SEM results. However, at this point, a quantitative correlation between the structural/morphologic parameters and the dielectric parameters variations is not possible due to the non-quantifiable structural evolution of the composite bulk structuring with increased graphene concentration.

Compared to existing literature results, Gaska et al. [23,24] studied the dielectric constant variation with graphene nanoplatelets concentration for LDPE-based composite materials prepared by extrusion method and reported an opposite variation, namely significant reduction of the dielectric constant of LDPE, from 2.8 to 1.2, in the frequency range of 10 Hz–0.1 MHz. No other studies regarding alike composite materials were found in the scientific databases. On the other hand, there are reports regarding other polymers where an enhancement of the dielectric constant with increasing graphene concentration was observed [25,26]. For a better understanding, the observed behavior, is currently under further investigation, in correlation with theoretical studies, this work limited to the investigation of the basic characteristics and their correlation with the respective dielectric properties. In any case, one possible explanation could be the fact that the degree of interfacial polarization greatly determines the dielectric properties of a medium. This indicates that introducing more interfaces can result in more probability of interfacial polarization and consequently, the dielectric response has more probability to be enhanced.

## 4. Conclusions

GnP/LDPE composite materials with various graphene concentrations were fabricated using the melting method and were characterized using SEM, XRD and Raman spectroscopy and microscopy in order to understand the formation of the composites and the evolution of structural and morphological characteristics with increasing graphene content. SEM characterization showed that graphene concentrations up to 2.5% lead to more compact structuring, while larger concentrations promote the random formation of a kind of “graphene bubbles”. XRD characterization showed that mean crystallite size increases as the graphene concentration increases, with a value of 26.1 nm at a concentration of 2%, this becoming 40.4 nm at 5%. Moreover, the increasing of the graphene concentration within the polymer matrix indicate an expansion of crystal interplanar distance of the embedded graphene. Regarding Raman characteristic signals of the composite regions, these appeared to increase in intensity as the graphene concentration increases, revealing the formation of a composite material and not a simple component mixing. The dielectric constants of the nanocomposites presented an increased trend, proportional with graphene increased concentration; however, it was strongly affected by the morphology evolution of the material. The random formation of “graphene bubbles” could induce the formation of conductive tunnels within the samples, this phenomenon being encountered at specimens with concentration of 4.5% and 5%, a behavior requiring further studies.
